# 2-[(6-Nitro-1,3-benzodioxol-5-yl)methyl­idene]malononitrile

**DOI:** 10.1107/S1600536811049816

**Published:** 2011-11-30

**Authors:** S. Karthikeyan, K. Sethusankar, Anthonisamy Devaraj, Manickam Bakthadoss

**Affiliations:** a Department of Physics, RKM Vivekananda College (Autonomous), Chennai 600 004, India; b Department of Organic Chemistry, University of Madras, Maraimalai Campus, Chennai 600 025, India

## Abstract

In the title compound, C_11_H_5_N_3_O_4_, the nitro group is rotated by 29.91 (16)° out of the plane of the adjacent aryl ring. The 1,3-benzodioxole ring is nearly planar, with a maximium deviation of 0.0562 (10) Å. The dioxolene ring adopts an envelope conformation on the O—C—O C atom. In the crystal, mol­ecules are linked *via* C—H⋯O inter­actions, resulting in *R*_2_^2^(6) and *R*_2_^2^(12) graph-set motifs.

## Related literature

For applications of malononitrile derivatives, see: Brimblecombe *et al.* (1972 ▶). For related structure, see: Loghmani–Khouzani *et al.* (2009 ▶). For comparison of mol­ecular dimensions, see: Allen *et al.* (1987 ▶). For puckering parameters, see: Cremer & Pople (1975 ▶). For graph–set motif notations, see: Bernstein *et al.* (1995 ▶). 
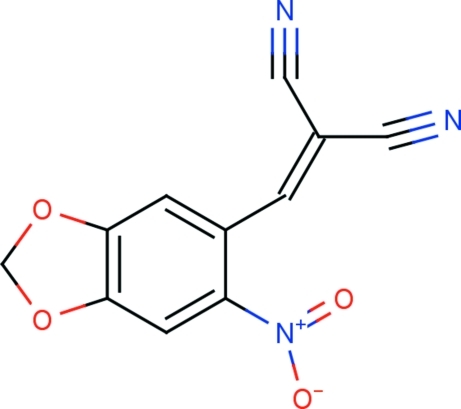


## Experimental

### 

#### Crystal data

C_11_H_5_N_3_O_4_
*M* *_r_* = 243.18
Triclinic, 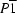

*a* = 7.0953 (2) Å
*b* = 8.8847 (3) Å
*c* = 9.2212 (3) Å
α = 84.470 (2)°
β = 67.634 (2)°
γ = 78.874 (2)°
*V* = 527.30 (3) Å^3^
*Z* = 2
Mo *K*α radiation
μ = 0.12 mm^−1^
*T* = 295 K
0.30 × 0.28 × 0.25 mm

#### Data collection

Bruker Kappa APEXII CCD diffractometer
13806 measured reflections
3494 independent reflections
2700 reflections with *I* > 2σ(*I*)
*R*_int_ = 0.025

#### Refinement

*R*[*F* ^2^ > 2σ(*F* ^2^)] = 0.048
*wR*(*F* ^2^) = 0.142
*S* = 1.03
3494 reflections
163 parameters
H-atom parameters constrained
Δρ_max_ = 0.28 e Å^−3^
Δρ_min_ = −0.30 e Å^−3^

### 

Data collection: *APEX2* (Bruker, 2008 ▶); cell refinement: *SAINT* (Bruker, 2008 ▶); data reduction: *SAINT*; program(s) used to solve structure: *SHELXS97* (Sheldrick, 2008 ▶); program(s) used to refine structure: *SHELXL97* (Sheldrick, 2008 ▶); molecular graphics: *ORTEP-3* (Farrugia, 1997 ▶) and *Mercury* (Macrae *et al.* 2008 ▶); software used to prepare material for publication: *SHELXL97* and *PLATON* (Spek, 2009 ▶).

## Supplementary Material

Crystal structure: contains datablock(s) global, I. DOI: 10.1107/S1600536811049816/rk2314sup1.cif

Structure factors: contains datablock(s) I. DOI: 10.1107/S1600536811049816/rk2314Isup2.hkl

Supplementary material file. DOI: 10.1107/S1600536811049816/rk2314Isup3.cml

Additional supplementary materials:  crystallographic information; 3D view; checkCIF report

## Figures and Tables

**Table 1:** Hydrogen-bond geometry (Å, °)

*D*—H⋯*A*	*D*—H	H⋯*A*	*D*⋯*A*	*D*—H⋯*A*
C1—H1*B*⋯O1^i^	0.97	2.53	3.2692 (16)	133
C8—H8⋯O4^ii^	0.93	2.52	3.3640 (17)	152

Symmetry codes: (i) 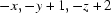; (ii) 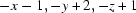.

**Fig. 1.**
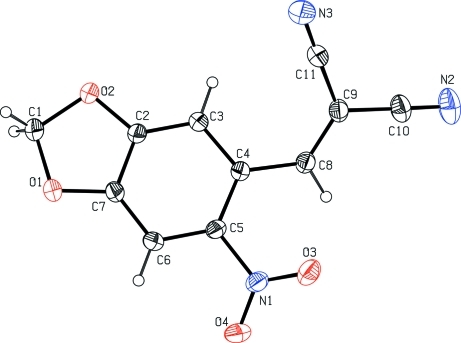
The molecular structure of the title compound with the atom numbering scheme, displacement ellipsoids are drawn at 30% probability level. H atoms are present as small spheres of arbitary radius.

**Fig. 2.**
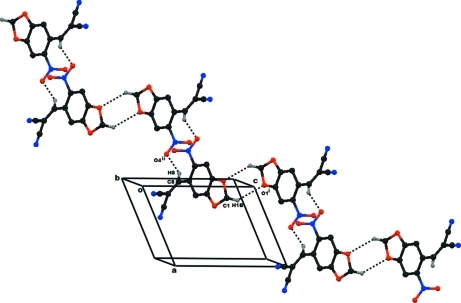
The packing arrangement of the title compound viewed down a axis. The dashed lines indicate C—H···O intermolecular interactions, which forms R22(6) and R22(12) centrosymmetric dimers. The symmetry codes: (i) -x, 1-y, 2-z; (ii) -1-x, 2-y, 1-z.

## supplementary crystallographic information

### Comment

The malononitrile derivative is used to investigate a variety of possible pharmacological effects when administered by various routes to whole animals and when applied to isolated organs and tissues (Brimblecombe *et al.*, 1972). Also, it is a component of "tear gas" commonly reffered as CS gas, which is used as a riot control agent.

In the title compound C_11_H_5_N_3_O_4_, the benzodioxole ring is nearly planar with a maximum deviation 0.0562Å for the atom O2. The O═N═O angle is much larger than the ideal tetrahedral or trigonal values, respectively, doubtless as a consequence of the substantial negative charge on the paired O atoms. The bond lengths C9—C10 = 1.4388 (16)Å and C9—C11 = 1.4342 (16)Å is significantly shorter than the expected value for a C—C single bond because of conjugation effects.

In the dioxole ring C1/O2/C2/C7/O1, the deviation of atom C1 is -0.0724 (16)Å. The dioxole ring adopts a *envelope* conformation on C1 with puckering parameters (Cremer & Pople, 1975): *Q*_2_ = 0.1145 (13)Å and φ_2_ = 36.7 (6)°. The malononitrile group (C9—C10≡N2) and (C9—C11≡N3) is almost linear, with the angle around central carbon atoms C10 and C11 being 179.14 (15)° and 179.09 (15)° respectively.

The values of the torsion angles C5–C4–C8–C9 = -154.85 (11)° and C4–C5–N1–O4 = -151.10 (12)° indicates that the conformation of molecule is (-)*anti*–periplanar. The nitro group is not co–planar to the benzodioxole ring to which it is attached, making a dihedral angle of 29.76 (4)°. The benzodioxole unit is oriented at a dihedral angle of 36.90 (4)° with respect to the malononitrile group. The triple bond distances C10≡N2 and C11≡N3 are in agreement with the literature values (1.138 (7)Å; Allen *et al.*, 1987). The title compound exhibits structural similarities with the already reported related structures (Loghmani–Khouzani *et al.*, 2009).

The crystal packing is stabilized by non–classical intermolecular C—H···O interactions. The molecules are linked into centrosymmetric dimers. Atom C1 acts as a donor to dioxole O1^i^, so forming an *R*^2^_2_(6) graph–set motif and atom C8 acts as a donor to nitro group O4^ii^ at forming an *R*^2^_2_(12) graph–set motif (Bernstein, *et al.*, 1995). Symmetry codes: (i) -*x*, 1-*y*, 2-*z*; (ii) -1-*x*, 2-*y*, 1-*z*).

### Experimental

To a solution of malononitrile (0.082 g, 1.24 mmol) in dichloromethane (5 ml), pyrrolidine (0.073 g, 1.03 mmol) was added and stirred well for 10 minutes. To this solution 6–nitrobenzo[*d*][1,3]dioxole–5–carbaldehyde (0.2 g, 1.03 mmol) was added and stirring was continued for 12 h. After the completion of the reaction as evidenced by *TLC*, the reaction mixture was poured into 2 *N* HCl solution (10 ml) and extracted using 25 ml of dichloromethane. The organic layer thus obtained was concentrated under reduced pressure. Column purification (silica gel, mesh size: 60–120) of the crude mixture using 15% ethyl acetate in hexanes successfully provided the desired 2–((6–nitrobenzo[*d*][1,3]dioxol–5–yl)methylene)malononitrile in 90% yield (0.23 g).

### Refinement

The hydrogen atoms were placed in calculated positions with C—H = 0.93Å to 0.97Å and refined in the riding model with fixed isotropic displacement parameters: *U*_iso_(H) = 1.2*U*_eq_(C) for aromatic and methylene groups.

### Crystal data

**Table:**

C_11_H_5_N_3_O_4_	*Z* = 2
*M**_r_* = 243.18	*F*(000) = 248
Triclinic, *P*1	*D*_x_ = 1.532 Mg m^−^^3^
Hall symbol: -P 1	Mo *K*α radiation, λ = 0.71073 Å
*a* = 7.0953 (2) Å	Cell parameters from 3494 reflections
*b* = 8.8847 (3) Å	θ = 1.0–31.6°
*c* = 9.2212 (3) Å	µ = 0.12 mm^−^^1^
α = 84.470 (2)°	*T* = 295 K
β = 67.634 (2)°	Block, yellow
γ = 78.874 (2)°	0.30 × 0.28 × 0.25 mm
*V* = 527.30 (3) Å^3^	

### Data collection

**Table:**

Bruker Kappa APEXII CCD diffractometer	2700 reflections with *I* > 2σ(*I*)
Radiation source: fine–focus sealed tube	*R*_int_ = 0.025
graphite	θ_max_ = 31.6°, θ_min_ = 2.3°
ω scans	*h* = −10→10
13806 measured reflections	*k* = −12→12
3494 independent reflections	*l* = −13→13

### Refinement

**Table:**

Refinement on *F*^2^	Primary atom site location: structure-invariant direct methods
Least-squares matrix: full	Secondary atom site location: difference Fourier map
*R*[*F*^2^ > 2σ(*F*^2^)] = 0.048	Hydrogen site location: inferred from neighbouring sites
*wR*(*F*^2^) = 0.142	H-atom parameters constrained
*S* = 1.03	*w* = 1/[σ^2^(*F*_o_^2^) + (0.0779*P*)^2^ + 0.0833*P*] where *P* = (*F*_o_^2^ + 2*F*_c_^2^)/3
3494 reflections	(Δ/σ)_max_ < 0.001
163 parameters	Δρ_max_ = 0.28 e Å^−^^3^
0 restraints	Δρ_min_ = −0.30 e Å^−^^3^

### Special details

**Table:**

Geometry. All s.u.'s (except the s.u. in the dihedral angle between two l.s. planes) are estimated using the full covariance matrix. The cell s.u.'s are taken into account individually in the estimation of s.u.'s in distances, angles and torsion angles; correlations between s.u.'s in cell parameters are only used when they are defined by crystal symmetry. An approximate (isotropic) treatment of cell s.u.'s is used for estimating s.u.'s involving l.s. planes.
Refinement. Refinement of *F*^2^ against ALL reflections. The weighted *R*–factor *wR* and goodness of fit *S* are based on *F*^2^, conventional *R*–factors *R* are based on *F*, with *F* set to zero for negative *F*^2^. The threshold expression of *F*^2^ > σ(*F*^2^) is used only for calculating *R*–factors(gt) *etc*. and is not relevant to the choice of reflections for refinement. *R*–factors based on *F*^2^ are statistically about twice as large as those based on *F*, and *R*–factors based on ALL data will be even larger.

### Fractional atomic coordinates and isotropic or equivalent isotropic displacement parameters (Å^2^)

**Table:**

	*x*	*y*	*z*	*U*_iso_*/*U*_eq_	
C1	0.1956 (2)	0.45131 (18)	0.79855 (15)	0.0499 (3)	
H1A	0.2553	0.3433	0.7969	0.060*	
H1B	0.2182	0.4995	0.8792	0.060*	
C2	0.13009 (16)	0.60560 (13)	0.61230 (12)	0.0335 (2)	
C3	0.13982 (17)	0.70725 (13)	0.48999 (12)	0.0345 (2)	
H3	0.2663	0.7286	0.4190	0.041*	
C4	−0.04642 (16)	0.77900 (12)	0.47404 (12)	0.0309 (2)	
C5	−0.23081 (16)	0.74097 (12)	0.58573 (13)	0.0331 (2)	
C6	−0.24088 (17)	0.63890 (13)	0.71134 (13)	0.0373 (2)	
H6	−0.3660	0.6171	0.7842	0.045*	
C7	−0.05588 (18)	0.57270 (13)	0.72113 (12)	0.0351 (2)	
C8	−0.04545 (17)	0.90159 (12)	0.35650 (13)	0.0347 (2)	
H8	−0.1612	0.9786	0.3818	0.042*	
C9	0.10476 (19)	0.91503 (13)	0.21576 (14)	0.0386 (2)	
C10	0.0900 (2)	1.05218 (15)	0.12130 (16)	0.0478 (3)	
C11	0.2814 (2)	0.79907 (17)	0.14691 (15)	0.0491 (3)	
N1	−0.42888 (15)	0.80802 (11)	0.57275 (13)	0.0417 (2)	
N2	0.0804 (3)	1.16093 (16)	0.04706 (18)	0.0707 (4)	
N3	0.4220 (2)	0.7081 (2)	0.09043 (17)	0.0760 (5)	
O1	−0.02108 (14)	0.46924 (11)	0.82958 (10)	0.0483 (2)	
O2	0.28869 (13)	0.52328 (11)	0.64860 (10)	0.0457 (2)	
O3	−0.43369 (15)	0.84163 (11)	0.44228 (12)	0.0507 (3)	
O4	−0.58153 (15)	0.82535 (14)	0.69255 (14)	0.0674 (4)	

### Atomic displacement parameters (Å^2^)

**Table:**

	*U*^11^	*U*^22^	*U*^33^	*U*^12^	*U*^13^	*U*^23^
C1	0.0424 (7)	0.0658 (8)	0.0332 (6)	0.0027 (6)	−0.0132 (5)	0.0108 (5)
C2	0.0307 (5)	0.0392 (5)	0.0288 (5)	−0.0007 (4)	−0.0115 (4)	−0.0001 (4)
C3	0.0298 (5)	0.0412 (5)	0.0304 (5)	−0.0058 (4)	−0.0101 (4)	0.0036 (4)
C4	0.0308 (5)	0.0307 (5)	0.0304 (5)	−0.0039 (4)	−0.0114 (4)	0.0004 (4)
C5	0.0279 (5)	0.0323 (5)	0.0367 (5)	−0.0011 (4)	−0.0111 (4)	−0.0009 (4)
C6	0.0306 (5)	0.0396 (6)	0.0343 (5)	−0.0040 (4)	−0.0056 (4)	0.0033 (4)
C7	0.0364 (5)	0.0372 (5)	0.0272 (5)	−0.0026 (4)	−0.0094 (4)	0.0024 (4)
C8	0.0349 (5)	0.0324 (5)	0.0388 (5)	−0.0057 (4)	−0.0167 (4)	0.0030 (4)
C9	0.0410 (6)	0.0401 (6)	0.0386 (6)	−0.0114 (5)	−0.0190 (5)	0.0086 (4)
C10	0.0602 (8)	0.0460 (7)	0.0449 (7)	−0.0211 (6)	−0.0253 (6)	0.0122 (5)
C11	0.0415 (7)	0.0632 (8)	0.0361 (6)	−0.0077 (6)	−0.0108 (5)	0.0109 (5)
N1	0.0309 (5)	0.0360 (5)	0.0550 (6)	−0.0031 (4)	−0.0153 (4)	0.0051 (4)
N2	0.1052 (13)	0.0544 (7)	0.0656 (9)	−0.0313 (8)	−0.0436 (9)	0.0243 (6)
N3	0.0539 (8)	0.0989 (12)	0.0516 (8)	0.0105 (8)	−0.0062 (6)	0.0049 (8)
O1	0.0414 (5)	0.0590 (6)	0.0362 (4)	−0.0034 (4)	−0.0116 (4)	0.0164 (4)
O2	0.0336 (4)	0.0603 (6)	0.0375 (4)	0.0003 (4)	−0.0141 (3)	0.0121 (4)
O3	0.0472 (5)	0.0499 (5)	0.0639 (6)	−0.0064 (4)	−0.0329 (5)	0.0056 (4)
O4	0.0323 (5)	0.0746 (7)	0.0705 (7)	0.0072 (5)	−0.0034 (5)	0.0157 (6)

### Geometric parameters (Å, °)

**Table:**

C1—O1	1.4319 (17)	C5—N1	1.4614 (14)
C1—O2	1.4334 (15)	C6—C7	1.3635 (15)
C1—H1A	0.9700	C6—H6	0.9300
C1—H1B	0.9700	C7—O1	1.3525 (13)
C2—O2	1.3547 (13)	C8—C9	1.3420 (16)
C2—C3	1.3641 (15)	C8—H8	0.9300
C2—C7	1.3850 (16)	C9—C11	1.4342 (19)
C3—C4	1.4068 (14)	C9—C10	1.4388 (16)
C3—H3	0.9300	C10—N2	1.1355 (18)
C4—C5	1.4016 (15)	C11—N3	1.138 (2)
C4—C8	1.4597 (14)	N1—O4	1.2145 (15)
C5—C6	1.3887 (15)	N1—O3	1.2230 (14)
			
O1—C1—O2	107.04 (9)	C7—C6—H6	122.1
O1—C1—H1A	110.3	C5—C6—H6	122.1
O2—C1—H1A	110.3	O1—C7—C6	128.09 (10)
O1—C1—H1B	110.3	O1—C7—C2	110.03 (10)
O2—C1—H1B	110.3	C6—C7—C2	121.87 (10)
H1A—C1—H1B	108.6	C9—C8—C4	126.91 (10)
O2—C2—C3	128.07 (10)	C9—C8—H8	116.5
O2—C2—C7	109.65 (9)	C4—C8—H8	116.5
C3—C2—C7	122.28 (10)	C8—C9—C11	124.80 (11)
C2—C3—C4	118.30 (10)	C8—C9—C10	119.43 (12)
C2—C3—H3	120.9	C11—C9—C10	115.74 (11)
C4—C3—H3	120.9	N2—C10—C9	179.14 (15)
C5—C4—C3	117.50 (9)	N3—C11—C9	179.09 (15)
C5—C4—C8	121.96 (9)	O4—N1—O3	123.24 (11)
C3—C4—C8	120.12 (9)	O4—N1—C5	118.09 (11)
C6—C5—C4	124.20 (10)	O3—N1—C5	118.67 (10)
C6—C5—N1	115.70 (10)	C7—O1—C1	105.81 (9)
C4—C5—N1	120.10 (10)	C2—O2—C1	105.89 (9)
C7—C6—C5	115.83 (10)		
			
O2—C2—C3—C4	179.32 (11)	C3—C2—C7—C6	0.79 (18)
C7—C2—C3—C4	−0.78 (17)	C5—C4—C8—C9	−154.85 (11)
C2—C3—C4—C5	0.01 (16)	C3—C4—C8—C9	32.75 (16)
C2—C3—C4—C8	172.74 (10)	C4—C8—C9—C11	8.72 (19)
C3—C4—C5—C6	0.80 (17)	C4—C8—C9—C10	−173.31 (10)
C8—C4—C5—C6	−171.78 (10)	C6—C5—N1—O4	29.91 (16)
C3—C4—C5—N1	−178.10 (9)	C4—C5—N1—O4	−151.10 (12)
C8—C4—C5—N1	9.32 (16)	C6—C5—N1—O3	−148.95 (11)
C4—C5—C6—C7	−0.81 (17)	C4—C5—N1—O3	30.04 (15)
N1—C5—C6—C7	178.14 (10)	C6—C7—O1—C1	−173.03 (12)
C5—C6—C7—O1	−179.34 (11)	C2—C7—O1—C1	7.55 (14)
C5—C6—C7—C2	0.01 (17)	O2—C1—O1—C7	−12.15 (14)
O2—C2—C7—O1	0.17 (14)	C3—C2—O2—C1	172.11 (12)
C3—C2—C7—O1	−179.75 (10)	C7—C2—O2—C1	−7.80 (14)
O2—C2—C7—C6	−179.29 (10)	O1—C1—O2—C2	12.26 (14)

### Hydrogen-bond geometry (Å, °)

**Table:**

*D*—H···*A*	*D*—H	H···*A*	*D*···*A*	*D*—H···*A*
C1—H1B···O1^i^	0.97	2.53	3.2692 (16)	133
C8—H8···O4^ii^	0.93	2.52	3.3640 (17)	152

Symmetry codes: (i) −*x*, −*y*+1, −*z*+2; (ii) −*x*−1, −*y*+2, −*z*+1.
